# Structural and biochemical characterization of arabinose nucleosides as inhibitors of the SARS-CoV-2 polymerase

**DOI:** 10.1063/4.0001152

**Published:** 2025-10-27

**Authors:** Ziyang Xiao, Thomas K. Anderson, Robert N. Kirchdoerfer

**Affiliations:** University of Wisconsin Madison, Madison, WI, USA

## Abstract

Severe acute respiratory syndrome coronavirus 2 (SARS-CoV-2) relies on its RNA-dependent RNA polymerase (RdRP) for viral genome replication and transcription, making it a critical target for antiviral drug development. While existing nucleotide analog inhibitors have shown therapeutic efficacy, their clinical application is hindered by limited potency, off-target effects, and the development of resistance. This underscores the urgent need for novel therapeutic strategies.

In this study, we examine arabinose nucleotide triphosphates (araNTP) as potential inhibitors of the SARS-CoV-2 RdRP. Using biochemical assays, we demonstrate that araNTPs effectively bind to the active site of RdRP, resulting in a strong stalling of subsequent nucleotide incorporation. We attribute the inhibition to the C-2’ endo sugar pucker of araNTP, which pauses next incorporation of the subsequent nucleotide. We used single particle cryo-EM to determine high-resolution structures of araCTP and araUTP incorporated substrates bound to the SARS-CoV-2 RdRp. These structures reveal detailed active site interactions indicating key hydrogen-bonding networks for araNTP incorporation.

Our work shows that araNTPs are potential compounds for new antiviral therapies. This work has the potential to introduce a novel therapeutic option for the treatment of coronavirus disease.

